# Transcriptome analysis of filling stage seeds among three buckwheat species with emphasis on rutin accumulation

**DOI:** 10.1371/journal.pone.0189672

**Published:** 2017-12-20

**Authors:** Jia Gao, Tingting Wang, Minxuan Liu, Jing Liu, Zongwen Zhang

**Affiliations:** 1 The Institute of Crop Science, Chinese Academy of Agricultural Sciences, Beijing, China; 2 School of Biology and Biological Engineering, South China University of Technology, Guangzhou, China; 3 China Office of Biodiversity International, Beijing, China; University of Vigo, SPAIN

## Abstract

Buckwheat is an important minor crop with pharmaceutical functions due to rutin enrichment in the seed. Seeds of common buckwheat cultivars (*Fagopyrum esculentum*, Fes) usually have much lower rutin content than tartary buckwheat (*F*. *tartaricum*, Ft). We previously found a wild species of common buckwheat (*F*. *esculentum* ssp. ancestrale, Fea), with seeds that are high in rutin, similar to Ft. In the present study, we investigated the mechanism by which rutin production varies among different buckwheat cultivars, Fea, a Ft variety (Xide) and a Fes variety (No.2 Pingqiao) using RNA sequencing of filling stage seeds. Sequencing data generated approximately 43.78-Gb of clean bases, all these data were pooled together and assembled 180,568 transcripts, and 109,952 unigenes. We established seed gene expression profiles of each buckwheat sample and assessed genes involved in flavonoid biosynthesis, storage proteins production, CYP450 family, starch and sucrose metabolism, and transcription factors. Differentially expressed genes between Fea and Fes were further analyzed due to their close relationship than with Ft. Expression levels of flavonoid biosynthesis gene *FLS1* (Flavonol synthase 1) were similar in Fea and Ft, and much higher than in Fes, which was validated by qRT-PCR. This suggests that *FLS1* transcript levels may be associated with rutin accumulation in filling stage seeds of buckwheat species. Further, we explored transcription factors by iTAK, and multiple gene families were identified as being involved in the coordinate regulation of metabolism and development. Our extensive transcriptomic data sets provide a complete description of metabolically related genes that are differentially expressed in filling stage buckwheat seeds and suggests that FLS1 is a key controller of rutin synthesis in buckwheat species. FLS1 can effectively convert dihydroflavonoids into flavonol products. These findings provide a basis for further studies of flavonoid biosynthesis in buckwheat breeding to help accelerate flavonoid metabolic engineering that would increase rutin content in cultivars of common buckwheat.

## Introduction

Buckwheat is a minor crop that belongs to the eudicot family Polygonaceae, genus *Fagopyrum*. Although buckwheat seeds mainly accumulate starches, they also have a balanced amino acid content, making them a good substitute for main foods.Before the nineteenth century, buckwheat was planted in seasons with a poor major grain harvest due to its fast growth and short life span [1]. Nowadays, buckwheat is well known for its pharmaceutical potential to protect people from cardiovascular disease, diabetes, and cancers, which is predominantly attributed to its content of rutin, quercetin and other flavonols with antioxidant and anti-inflammatory bioactivities [2]. These characteristics have made this plant more popular as a healthy, yet traditional food crop, and demand for buckwheat products grows each year.

Buckwheat is mainly cultivated in China, Russia, and Ukraine and prevalent in Japan, Korea, India and Himalayan areas, like Nepal and Bhutan, as traditional crops [3–4]. *Fagopyrum* contains 23 species, with vastly different phenotypes. Two prevalently cultivated buckwheat species are *F*. *esculentum* (Fes; common buckwheat cultivar) *and F*. *tartaricum* (Ft; tartary buckwheat cultivar). Genetic and environmental factors, as well as their interactions, influence the metabolism and development of seeds over time [5–6]. Fes is commonly planted in vast plateaus, while Ft is cultivated in mountainous and high-altitude areas. Fes is self-incompatible with an easily dulled seed coat, whereas Ft is self-compatible with a tight seed coat. These species also differ in their morphology, flour flavor, and seed rutin content [7–9]. Common buckwheat seeds have better flavor and are easily processed making its cultivation more prevalent than tartary buckwheat. On the other hand, the lower rutin content in Fes seeds represents a crucial disadvantage to this species [1]. The abundant diversity of wild species germplasm gene resources makes it possible to improve the agricultural traits of cultivars [10]. However, the advantages and disadvantages between those two buckwheat cultivars could not be engineered by traditional hybridization. Due to limited information on the mechanism of rutin production, genetically engineering and breeding common buckwheat with high rutin content remains a significant challenge [11]. Besides, starch is one of the most important nutrients in buckwheat seeds[12]. Starch could be divided structurally into amylose and amylopectin two kinds of glucose polymers. Amylose is synthesized by granule-bound starch synthase and ADP-glucose pyrophosphorylase while amylopectin is synthesized by ADP-glucose pyrophosphorylase, soluble starch synthase (SS), starch branching enzyme, and starch debranching enzyme which includes isoamylase for the creation of normal branching patterns[13]. Sucrose plays dural roles in both storage and regulation of gene expression in plants. Sucrose is synthesized from glucose 1-phosphate by UDP glucose pyrophosphorylase, sucrose phosphate synthase and sucrose phosphatase. On the other side, the cleavage of sucrose is catalyzed by sucrose synthase in which process the substrate will produce glucose and fructose. The balance of synthesis of sucrose and starch in the cytoplasm and chloroplast respectively is regulated by many genes. However, studies focused on the starch and sucrose metabolism regulation of buckwheat species are still missed[14].

Next generation RNA sequencing (RNA-seq) enables us economic investigation of the molecular basis of complex plant metabolic processes. Efforts have been made to study flavonoid biosynthesis and processing via transcriptomic analysis. Chen and Li (2016) studied genes involved in the biosynthesis of epicatechin and catechin in the rhizomes of *F*. *dibotrys* and its mutant [15]. Yao *et al*. studied the flowers of different tartary buckwheat cultivars with different seed coat color and found that differentially expressed genes (DEG) involved in flavonoid synthesis may correlate with variations in rutin, quercetin, and kaempferol content [16].

**Fig 1:**
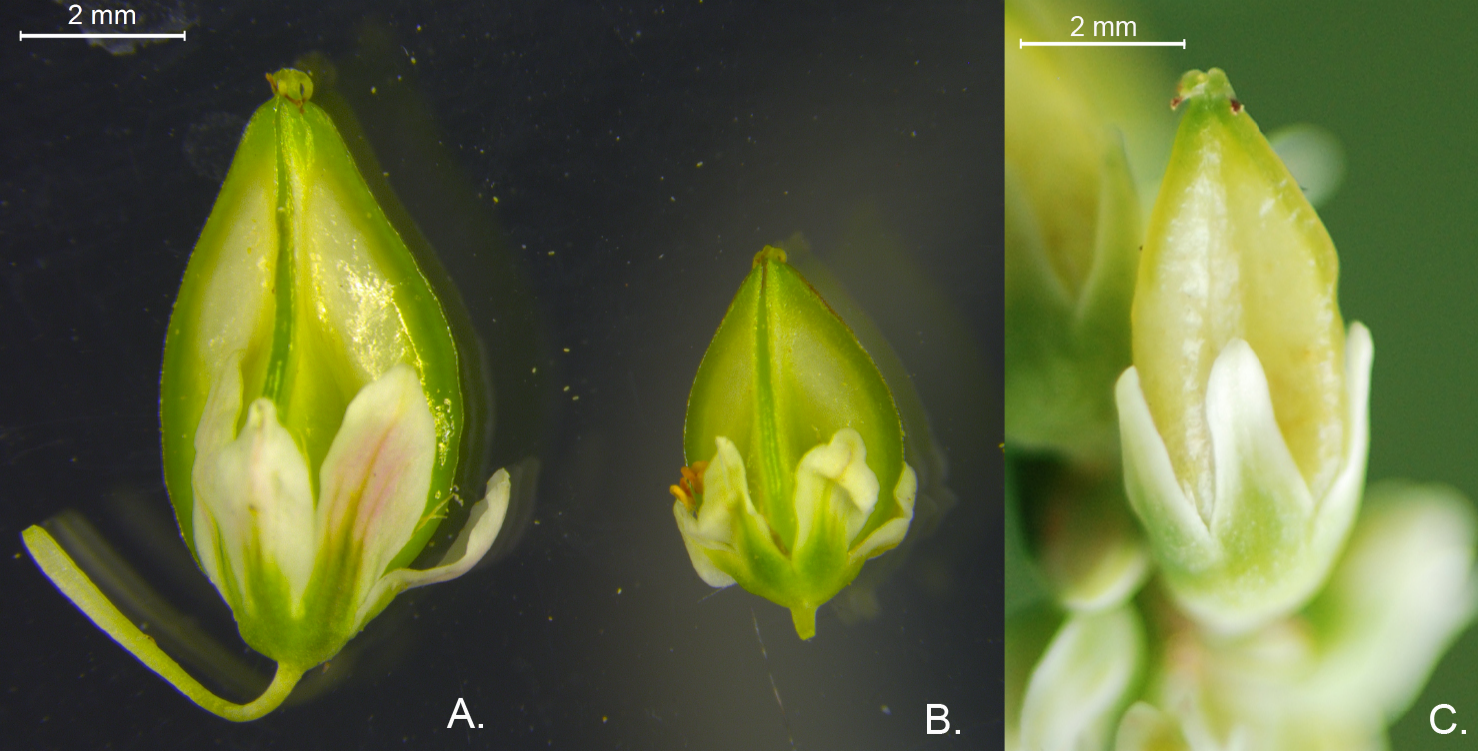
Buckwheat seeds in the filling stage. Comparison of filling-stage seeds of (A) common buckwheat (Fes), (B) a wild species of common buckwheat (Fea), and (C) tartary buckwheat (Ft).

Seeds are the primary storage tissue of buckwheat species and many nutritional quality-related investigations have reported. However, no studies have used transcriptome sequencing to investigate the variation in seed rutin accumulation differentiation among buckwheat species. Here, we collected a wild ancestral species of common buckwheat (*F*. *esculentum* ssp. *ancestrale*, Fea) from southwestern China, with smaller floral organs and seed size than cultivated species and seed rutin content that is similar to Ft (Fig 1). In the present work, we employed RNA-seq technology to identify genes involved in Fes, Fea and Ft metabolism, emphasizing elucidation of genes involved in rutin production and those differentially expressed among these buckwheat species.

## Materials and methods

### Plant material

Seeds of Ft (Xide), Fes (No.2 Pingqiao) and Fea (*F*. *esculentum* ssp. *ancestrale*) were conserved in the National Germplasm Resource Center of China. To investigate the biodiversity and comparative relationships, these species were sown in May 2014, and grown in the experimental field at Yanqing (Beijing, China; 115° 44’, 40° 47’, latitude 500 m). Fea and Fes were separately planted by nylon nets. We separated the developing buckwheat seeds into three different stages: 1–12 day after pollination (1–12 DAP), the filling stage (15–20 DAP), and the period just before the seed coat color started to change (20–30 DAP).

### Analysis of rutin in developing seeds by high performance liquid chromatography (HPLC)

Samples of the immature seeds were separately collected at three stages examined. In the first stage as the (1–12 DAP), the ovulary starts to enlarge until the top of the seed is equal to the petal tip. In the second stage (15–20 DAP), the seed top exceeds the petal tip till it is almost full length. In stage 3 (21-30DAP), the seed is filled with starch and still green in color. Seeds were separately freeze-dried for 48 h at -51°C, then ground into a fine powder. Powdered seed samples (100 mg each) were then dissolved in 1mL methanol and put into a 37°C water bath with ultrasonication for 1 h. After, these samples were centrifuged 5 min at room temperature and the supernatants filtrated with a 0.22 μM filter before loading 20 μL onto Ultimate LP-C18 column (4.6 × 250 mm, 5 μm; Welch, Jinhua, China) on a Shimadzu LC-2010A HPLC analyzer (Shimadzu, Kyoto, Japan). The column was held at 30°C, with a flow rate of 1 mL/min. Rutin standards were diluted into five gradients in pure filtered methanol and run with a mobile phase of methanol: water: acetic acid [5:92.5:2.5 (v: v: v) % for buffer A; 95:2.5:2.5 (v: v: v) % for buffer B]. Rutin was detected at 365 nm and identified according to the retention time and Ultra Violet spectra of standards. Linear gradient elution began with 100% buffer A and 0% buffer B, followed by 65% buffer B at 8 min, 80% buffer B for 3 min, then 100% Buffer A for 5 min. The elution curve and rutin concentration of each sample were recorded and calculated for three replicates.

### RNA extraction and cDNA library construction

The filling stage seeds were applied to RNA-seq. Fresh filling stage seeds were fast frozen and stored in liquid nitrogen until use. Total RNA from each sample was extracted using an RNeasy Plant kit (Qiagen, Valencia, USA). The purity of each RNA sample was checked with a Nano-Photometer spectrophotometer (IMPLEN, Munich, Germany), the integrity and concentration were determined using an Agilent Bioanalyzer 2100 system (Agilent Technologies, Santa Clara, USA) and Qubit 2.0 Fluorimeter (Life Technologies, South San Francisco, USA), respectively.

The plant mRNA was prepared to construct the cDNA library, and RNA-seq was carried out on an Illumina HiSeq^TM^2500 platform. In brief, mRNA was isolated from total RNA using oligodT magnetic beads. Short mRNA fragments (150–250 bp) were used as templates for first-strand cDNA synthesized with random hexamer primers and M-MuLV reverse transcriptase. Second-strand cDNA synthesis was carried out by adding dNTPs, RNase H, and DNA polymerase I. After purification, ends were repaired, adenine-base were added, and each end of the double-stranded cDNA fragments were ligated with Illumina-indexed adaptors. Properly sized fragments were selected and amplified by polymerase chain reaction (PCR) to generate templates. The cDNA templates were further enriched by amplification to generate the cDNA library. The cDNA library was then sequenced on an Illumina HiSeq™ 2500 platform using a paired-end pipeline.

### *De novo* transcriptome assembly

Raw reads were trimmed off adaptor sequences for quality control. Then, reads containing more than 5% vague nucleotides and those with low quality containing more than 50% bases with a *Q*-phred value ˂ 20 were eliminated; clean reads of samples were generated and used for RNA *de novo* assembly with Trinity program[version r20140413][17], and the longest transcripts were taken and defined as unigenes. All unigenes were arranged in descending order from the first unigene. When the assembled length covered half the total length of all unigenes, the length of the current unigenes was considered to be N50 statistics. When the assembled length covered 90% of the total length, the length of the current unigene was considered to be N90 statistics.

### Annotation and classification of the *de novo* transcriptome

BLASTx alignment (*E*˂10^−5^) between unigenes and protein databases was performed. The best alignment results were used to determine the sequence direction, coding regions and amino acid sequence. All unigenes were searched for homologous genes using BLAST and annotation against the NCBI Non-Redundant Protein (NR) and Nucleotide (NT) databases (http://www.ncbi.nlm.nih.gov/) using an *E*-value cut-off of 10^−5^. Unigene sequences were also aligned by BLASTx to various protein databases in the following order: Swiss-Prot (http://www.ebi.ac.uk/uniprot/), Gene Ontology (GO) [http://www.geneontology.org/], Pfam (http://pfam.janelia.org/), Eukaryotic Orthologous Groups (KOGs) [ftp://ftp.ncbi.nih.gov/pub/COG/KOG/], and Kyoto Encyclopedia of Genes and Genomes Ortholog database (KO) [http://www.genoe.jp/kegg/]. The unigenes were sorted to recover proteins with the most similarity to unigenes with putative functional annotations. GO terms at the second level were used to perform the GO annotation of unigenes which assigned into the biological process, molecular functions and cellular components classification. Unigene sequences were also aligned to the KOG database to predict and classify possible functions, and pathway assignments were provided according to the KEGG pathway database.

### Identification and annotation of seed metabolism relevant genes

A set of representative keywords was used to predict starch metabolism, flavonoid biosynthesis-related genes, and other metabolic relevant genes important for seed development based on BLAST annotation results. Each gene was individually searched by gene name and related gene regions that were conserved in other species were identified. To find the most important genes related to flavonoid and starch metabolic system functions, GO term and KEGG pathway information were also used.

### Quantitative reverse transcriptase-PCR (qRT-PCR)

A QuantiTect Reverse Transcription Kit (Qiagen, Valencia, USA) was used according to the manufacturer’s instructions to generate the first strand cDNA after extracting total RNA from samples subjected to RNA-seq. The expression levels of 11 flavonoid biosynthesis DEGs were confirmed by qRT-PCR with the resulting cDNA products as templates. Primer sets were designed from the coding region results of sequencing data. Conserved domains were chosen as the area without single nucleotide polymorphisms (SNPs) and other mutations which may affect the efficiency of amplification by resequencing and redesign. FastStart Universal SYBR Green Master Mix (10μL; Roche, Indianapolis, USA) was mixed with gene-specific primers (3μL), sterilized water (5μL), and the synthesized cDNA (2 μL) in a 20 μL total as the total reaction volume. Reactions were performed on an ABI 7300 Real-Time cycler (Applied Biosystems, San Francisco, USA). Preheated to 95°C for 10 min; the three-step program (50 cycles) began at 95°C for 15 s, followed by 60°C for 30 s, and then72°C for 30 s. The 2^-ΔΔCt^ method was used to calculate the relative expression level of each unigene and tartary buckwheat histone H3 was selected as the reference gene for normalization [18].

### Statistical analyses

Statistical analyses were performed with Excel (2016). The data are expressed as the means and standard deviations of three replications. One way ANOVA analysis of variance was used to compare the significant difference. We declared significance at an α level≤0.05 for all statistical analyses followed by Tukey's post hoc comparisons. A *P* < 0.05 (*) was considered statistically significant and *P* < 0.01 (**) was considered statistically extremely significant for all data.

## Results

### HPLC analysis of rutin content in developing buckwheat seeds

**Fig 2:**
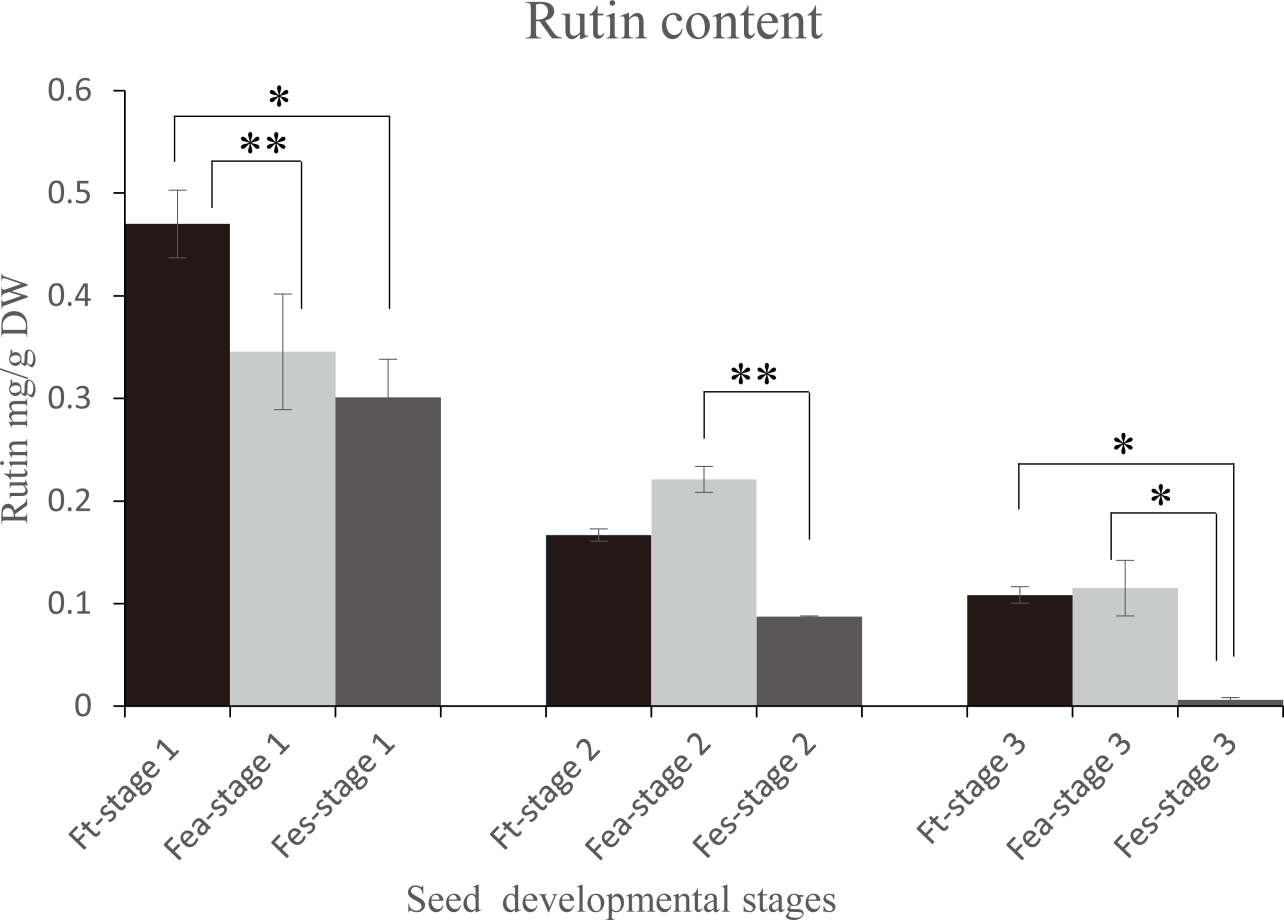
Comparison of rutin contents in buckwheat seeds at three different developmental stages (stage2: Filling stage). Values are expressed as the mean ± standard error of three replicates.

HPLC analysis was used to quantify the rutin content in developing Fea, Ft, and Fes seeds at three different stages of development (Fig 2). In all samples, changes in rutin content with growth were similar, reaching their highest value at the very beginning of seed development and decreasing with maturation. However, the absolute content varied by species. In the filling stage, rutin content in Fes decreased to half that in Ft, while levels in Fea were higher than Ft. In stage 3, the rutin content decreased to almost undetectable levels in Fes; rutin levels in Ft and Fea were similar and 10-times higher than in Fes.

### RNA-seq and *de novo* assembly in filling stage buckwheat seeds, classification and annotation

In order to assess the mechanism of rutin production, we performed RNA-seq on Fea, Ft, and Fes buckwheat seeds. Three cDNA libraries were constructed from fresh filling stage seeds. Sequencing of these libraries generated 29.74, 27.12 and 30.61 million clean reads (filtered from 30.35, 28.56, and 31.9 million raw reads respectively) with *Q*20 scores of 95.48%, 95.2%, and 95.09%, respectively, and the GC contents of these species were 45.98%, 47.15%, and 47.97%, respectively (S1 Table). These data were pooled together to create a comprehensive reference transcriptome, which was then used to facilitate analysis of differential gene expression and abundance in each sample. The clean reads were *de novo* assembled into 109,952 unigenes using Trinity [17] and had a mean length of 677 bp; the minimum length was controlled by a quality limitation to 201bp (S2 Table). The size distribution of these unigenes is shown in S1 Fig. The mapping rates of the buckwheat samples to the reference transcriptome are listed in S3 Table. The total number of reads for Fea, Fes, and Ft was 61,222,670, 59,483,078, and 54,249,377 bp, respectively, and the total number of mapped transcripts was 43,818,336 bp (71.58%), 45,390,194 bp (76.31%, and 39,048,665 bp (71.98%), respectively. The sequence database generated in this study is available on the NCBI database Short Read Archive under accession number SRP095982.

**Fig 3:**
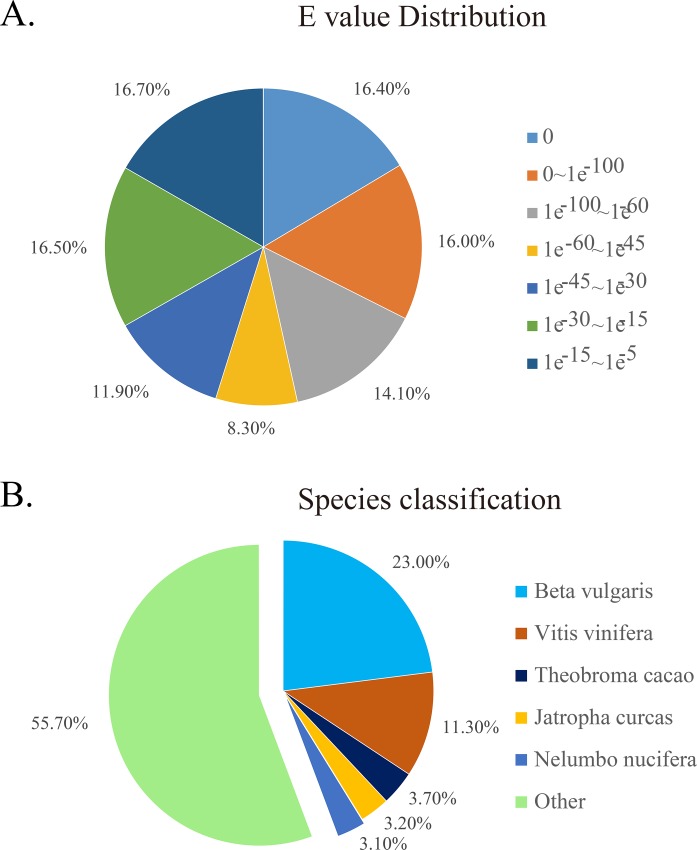
(A) The E-value distribution of unigenes. (B) Buckwheat species annotation distribution.

Unigenes were annotated by BLASTx (cutoff *E*-value 10^−5^) against seven public databases (NR, Pfam, Swiss-Prot, GO, NT, KO and KOG; Table 1). To sum up, 38,910 unigenes were annotated in NR (35.38%), whereas the distribution of annotated hits against other databases was: 29,235 (26.58%) with Swiss-Prot, 28,648(26.05%) for GO, 28,288 (25.72%) from Pfam, 16,699 (15.18%) with NT, 14,342 (13.04%) from KO, and 14,326 (13.02%) for KOG, we also did assembled each species transcriptomes (S4 Table) to check the uniform of annotated unigene sequence involved in flavonoid biosynthesis. Based on the NR annotation and *E*-value distribution, 34.3% of the mapped sequences showed significant homology (*E*<10^−30^) and 32.4% showed very significant homology (*E*<10^−100^) to the available plant sequences. Analysis of species annotation distribution showed that *Fagopyrum* spp. had the most matched homologous unigenes with *Beta vulgaris* (23%), followed by *Vitis vinifera* (11.3%), *Theobroma cacao* (3.7%), *Jatropha curcas* (3.2%), *Nelumbo nucifera* (3.1%), and others (55.7%) [Fig 3].

**Table 1:** Gene annotation and BLAST results against seven public databases.

	Number of unigenes	BLAST hits (%)
Annotated in NR Annotated in NT Annotated in KO Annotated in SwissProt Annotated in PFAM Annotated in GO Annotated in KOG Annotated in all databases Annotated in at least one Database Total Unigenes	38910 16699 14342 29235 28288 28648 14326 5615 44272 109952	35.38 15.18 13.04 26.58 25.72 26.05 13.02 5.1 40.26 100

**Fig 4:**
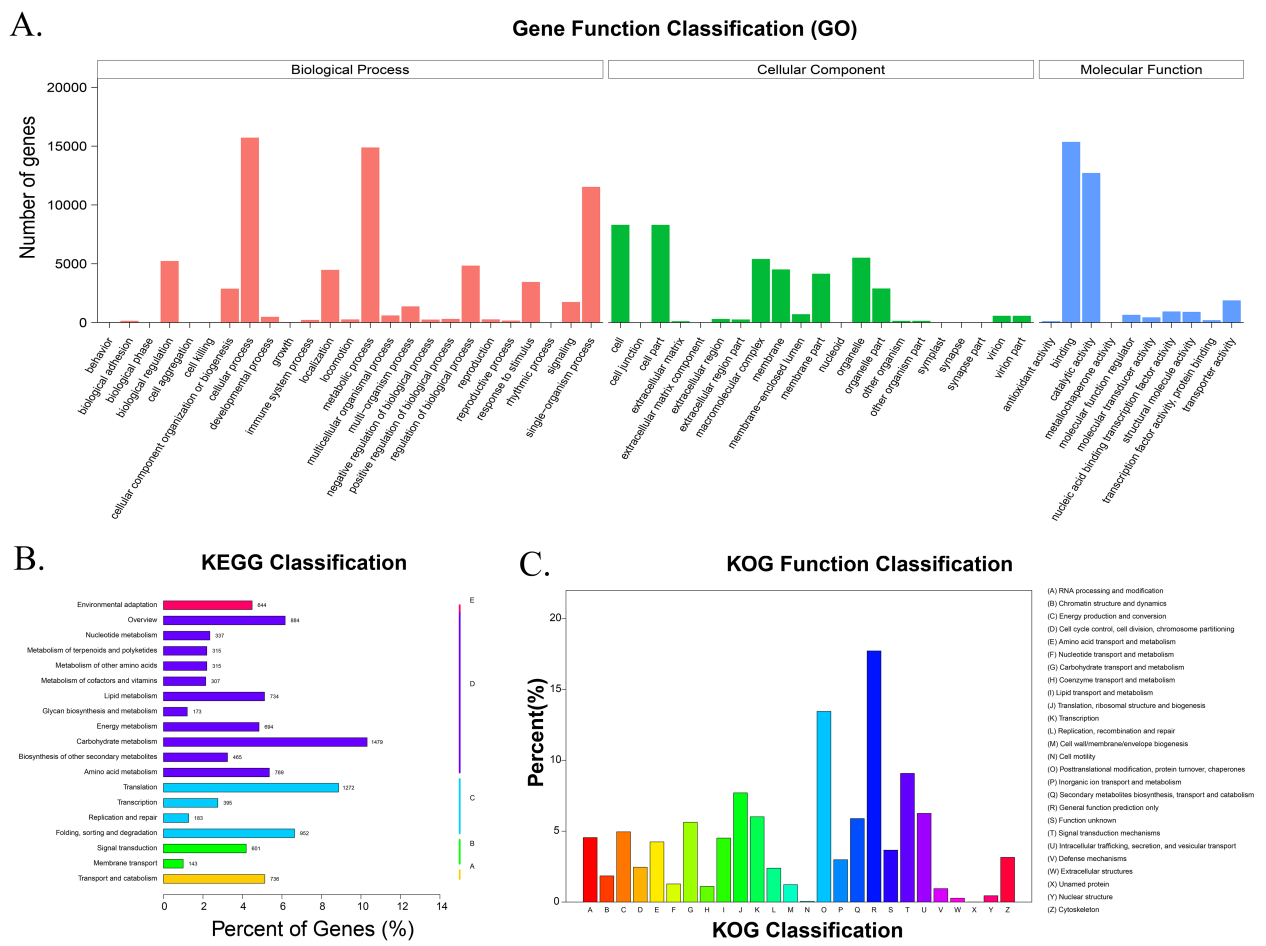
(A) GO annotation of all unigenes in filling-stage buckwheat seeds. Annotated sequences were catalogued into three major classes. (B)KEGG assignment of all unigenes. (C)KOG functional classifications.

*Fagopyrum* spp. expressed genes were functionally assigned with three primary GO terms related to biological processes, molecular function, and cellular components (Fig 4A). In general, 28,648 unigenes annotated in the GO database were sorted into 56 terms. Regarding the biological process: cellular (GO: 0009987; 15,720 unigenes) and metabolic (GO: 0008152; 14,887 unigenes) subclusters were predominant. Under the cell aggregation term (GO: 0098743), there was only 1 predicted unigene, the biological phase (GO: 0044848) contained 21 unigenes, and rhythmic processes (GO: 0048511) included 25 unigenes. Within the molecular function category, unigenes were primarily assigned to three major terms: binding (GO: 0005488; 15,360 unigenes), catalytic activity (GO: 0003824; 12,718 unigenes), and transporter activity (GO: 0005215; 1880 unigenes), whereas, the metallochaperone activity only contained 8 unigenes. Finally, for the cellular function term, 8307 unigenes clustered with cell function (GO: 0005623), 8304 with cell part (GO: 0044464), 5513 with organelle (GO: 0043226), and 13 with cell junctions (GO: 0030054).

**Fig 5:**
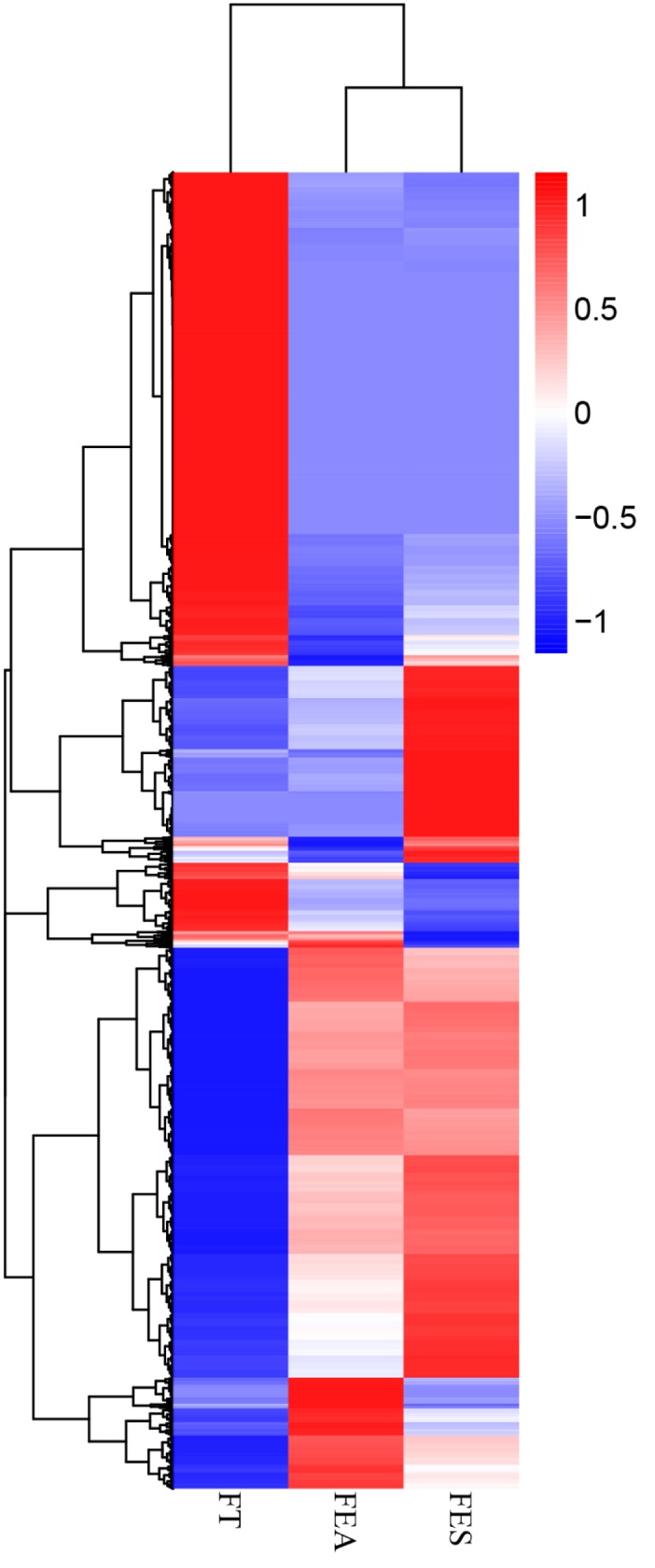
Heatmap of differentially expressed genes among three buckwheat species.

A total of 25,666 differentially expressed genes were detected among three *Fagopyrum* spp. libraries, of which 11,658, 24,335, and 2385 DEGs were predicted from Fea versus Ft, Ft versus Fes, and Fes versus Fea, respectively (Fig 5). *Q* <0.001 and the absolute value of log2 ratio ≥1 was used as the screening threshold for differentially expressed genes [19].

In 992 GO annotated DEGs, enrichment analysis showed that Fea versus Fes had 37 unigenes enriched in both heme (GO: 0020037) and tetrapyrrole (GO: 0046906) binding, under the molecular function term, oxidation-reduction processes (GO: 0055114) were enriched with 134 unigenes under the biological processes term (all adjusted with corrected *P* = 0.020565), and oxidoreductase activity was enriched with 131 unigenes (GO: 0016491; corrected *P =* 0.042052). Fea versus Fes had no down-regulated unigenes enriched in any GO terms, whereas upregulated genes were enriched in oxidoreductase activity (GO: 0016491; 87 unigenes), oxidation-reduction processes (GO: 0055114; 87 unigenes), carbohydrate metabolic processes (GO: 0005975; 53 unigenes), hydrolase activity on glycosyl bonds (GO: 0016798; 28 unigenes), hydrolase activity on *o*-glycosyl compounds (GO: 0004553; 27 unigenes), lignin catabolic processes (GO: 0046274; 3 unigenes), lignin metabolic processes (GO: 0009808; 3 unigenes), and phenylpropanoid metabolic processes (GO: 0046271; 3unigenes). Fea versus Ft was enriched with only one item, oxidoreductase activity (GO: 0016491; 580 unigenes). Fes versus Ft was enriched with organic acid (GO: 0016053; 268 unigenes), carboxylic acid (GO: 0046394; 268 unigenes), and small molecule (GO: 0044283; 341 unigenes) biosynthetic processes, (all with corrected *P-*value = 0.01274).

Gene interactions within filling stage *Fagopyrum* spp. seeds were explored with KEGG (Fig 4B). A total of 14,342 unigenes were mapped to plant canonical pathways and classified into five main pathways which included metabolism, genetic information processing, environmental information processing, cellular processes, and organismal systems. Those genes were further assigned to 130 pathways which were closely related to plant physiological activities in developing buckwheat seeds. Biosynthesis, assimilation, and degradation pathways were all involved: carbohydrate metabolism (1479 unigenes); translation (1272 unigenes); folding, sorting, and degradation (952 unigenes); amino acid metabolism (769 unigenes); transport and catabolism (736 unigenes); lipid metabolism (734 unigenes). Besides active carbohydrate, protein, and lipid metabolism, transportation was also important between the embryo and endosperm. Membrane transport was mapped with 143 unigenes, replication and repair with183 unigenes, and glycan biosynthesis and metabolism with 163 unigenes. These annotations might serve as a resource for elucidating the metabolic pathways and signaling networks in seeds which regulate metabolism in buckwheat. Except for the five main elements of metabolism, we were focused on the biosynthesis of secondary metabolites present in buckwheat seeds. Biosynthesis of other secondary metabolites were mapped with 465 unigenes: phenylpropanoids (ko00940; 259 unigenes, 55.7%);, flavonoids (ko00941; 92 unigenes,19.8%); terpenoid backbones (ko00900; 88unigenes, 18.9%); phenylalanine metabolism (ko00360;75unigenes,16.1%); stilbenoids, diarylheptanoids, and gingerol biosynthesis (ko00945; 71 unigenes,15.3%); flavones and flavonols (ko00944; 22unigenes, 4.7%); anthocyanins (ko00942;13 unigenes, 2.8%); indole alkaloids (ko00901; 5 unigenes,1.07%); isoflavonoids (ko00943; 4 unigenes,0.86%).

We also used KEGG assignments to classify the functions of the DEGs in pairwise comparisons among the filling stage seeds in the FT, FEA and FES. For the KEGG pathways, the specific enrichment of unigenes was observed for several pathways that were involved in flavonoids metabolic processes, such as phenylalanine metabolism (ko00360,19 unigenes), phenylpropanoid biosynthesis (ko00940,20 unigenes) and flavonoid biosynthesis (ko00941,9 unigenes), with multiple terms assigned to the same unigenes. When Fea was compared with Fes, enriched genes were involved in phenylpropanoid biosynthesis, flavonoid biosynthesis and alpha-linolenic acid metabolism (ko00592), ubiquinotne and other terpenoid-quinone biosynthesis (ko00130), tyrosine metabolism (ko00350), diterpenoid biosynthesis (ko00904), phenylalanine, tyrosine and tryptophan biosynthesis (ko00400). Fea compare to Ft was down regulated in ribosome biogenesis in eukaryotes (ko03008). Up-regulated unigenes in diterpenoid biosynthesis (ko00904), selenocompound metabolism (ko00450), Flavonoid biosynthesis (ko00941). Fes versus Ft was down regulated in fatty acid elongation (ko00062).

Eukaryotic transcripts were classified by the KOG database. KOG annotation was performed for the whole *Fagopyrum* spp. as showed in Fig 4C. The result showed that most of the genes (2539 unigenes) were clustered under the general function prediction category due to the lack of related genome under the Polygonaceae order. We found 1928 unigenes were categorized as posttranslational modifications, protein turnover, and chaperones, which illustrates active biosynthesis during seed development; 1301 unigenes were involved in signal transduction mechanism; 1104 unigenes were related to translation, ribosomal structure, and biogenesis; 897 unigenes were associated with intracellular trafficking, secretion, and vesicular transport; 862 unigenes were implicated in transcription;844 unigenes were involved in secondary metabolite biosynthesis, transport, and catabolism; 807 unigenes were related to carbohydrate transport and metabolism. Other genes involved in metabolism were divided into lipid transport and metabolism (647 unigenes), amino acid transport and metabolism (608 unigenes), inorganic ion transport (428 unigenes), coenzyme transport and metabolism (158 unigenes), and nucleotide transport and metabolism (183 unigenes).

### Flavonoid biosynthesis pathway expression profile

**Fig 6:**
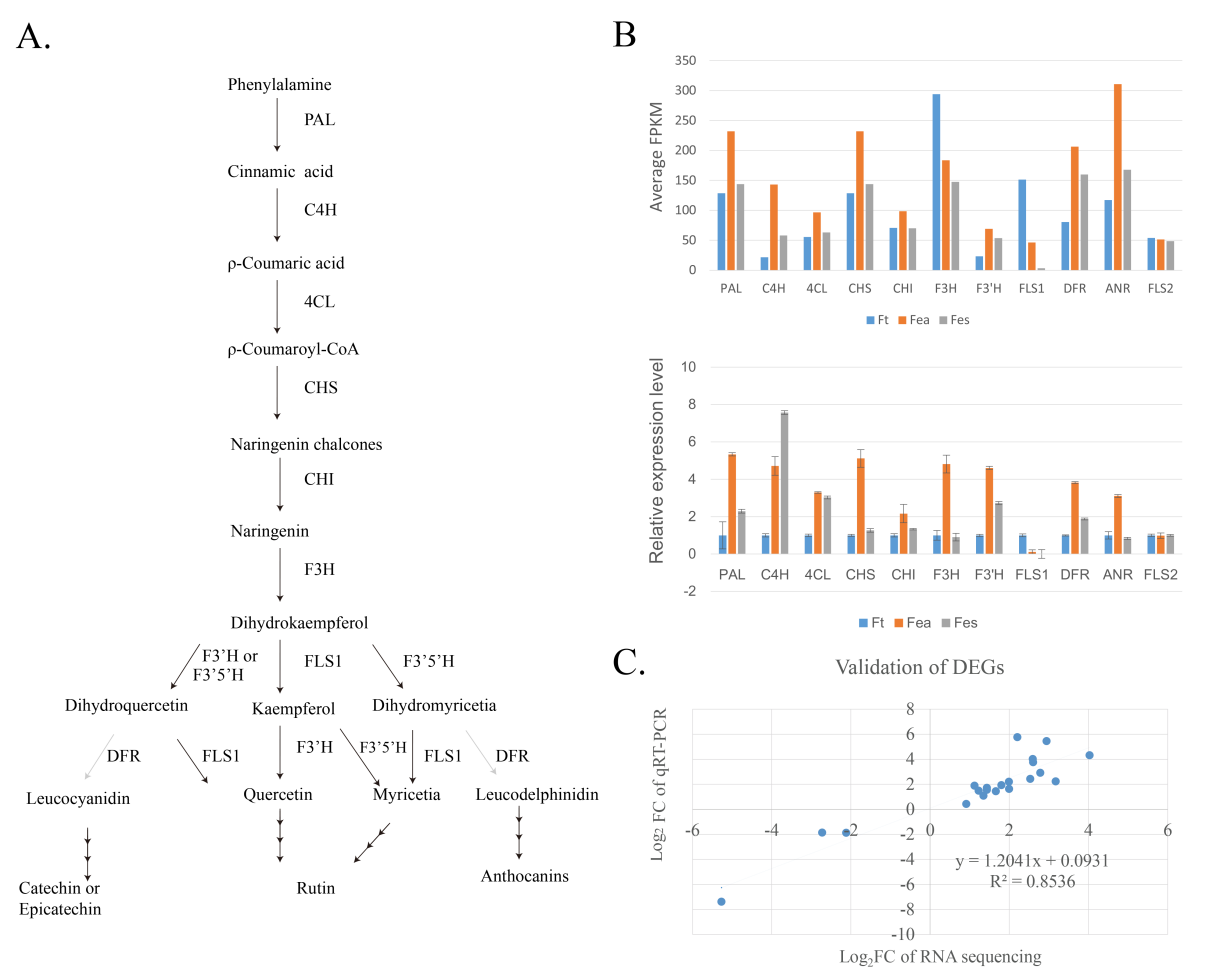
Gene expression profiles of flavonoids biosynthesis genes. (A) Scheme of the biosynthetic pathway of flavonoids. (B) Comparison of gene expression patterns involved in flavonoid biosynthesis in Ft, Fea, and Fes between RNA-seq FPKM and qRT-PCR data (error bar indicates standard deviation). (C) Validation of differentially expressed flavonoid biosynthesis-related genes.

Flavonoids are UltraViolet protectants in plants (Fig 6A). The biosynthesis pathway involved enzymes as follows: phenylalanine ammonia-lyase (c78103_g1, c76752_g1, c65505_g1, c65563_g1, c43581_g1, and c80029_g1), cinnamic acid 4-hydroxylase (c78622_g1, c65841_g1,), 4-coumarate-CoA-ligase (c76157_g1, c79904_g1, c75279_g1, c80099_g1, and c79681_g1), chalcone synthase (c70318_g1-5), chalcone isomerase (c67312_g1), flavonoid 3-hydroxylase (c74521_g1, c76122_g1), flavonoid 3’-hydroxylase (c75726_g2, c67918_g1, and c79262_g1), and flavonoid 3’,5’-hydroxylase (c79279_g1). Three isoforms of flavonol synthase were annotated in our unigenes: flavonol synthase 2 (c71512_g1), flavonol synthase 1 (c62983_g1), and flavonol synthase 5 (c62154_g1). The anthocyanins biosynthesisinvolved anthocyanidin synthase (c69216_g1), anthocyanidin reductase (c71792_g1), dihydroflavonol 4-reductase (c62371_g1 and c75529_g1), and leucoanthocyantin reductase (c73957_g1). The expression profiles of flavonoid biosynthesis pathway genes were evaluated by qRT-PCR using cDNA templates from the same samples of each buckwheat species (S5 Table). *PAL*, *4CL*, *CHS*, *CHI*, *F3’H*, *DFR*, and *ANR* were with the similar expression ratio with FPKM. C4H expression was slightly high in Fes. However, FLS2 expression showed no difference in the three species. *FLS1*, on the other hand, showed significant variation among these buckwheat species. In Ft, *FLS1* had the highest FPKM, followed by Fea and Fes (Fig 6B).

### DEGs involved in flavonoid biosynthesis and qRT-PCR validation

The RNA-seq gene expression level was screened using absolute value of log2 fold change >1 and the FPKM >1. qRT-PCR validation was performed based on the sequencing results. Primers were designed based on the alignment of the most conserved domain on the UniProt database (http://www.uniprot.org); histone H3 was used as the reference gene. We tested the expression of 11 DEGs involved in flavonoid biosynthesis with qRT-PCR to validate results using FPKM levels (Fig 6C). Based on log2-fold change measurements, the correlation between RNA-seq and qRT-PCR were evaluated (S5 Table). The results showed that the relative expression trends of these DEGs were significantly correlated to the data as those of RNA-seq (r^2^ = 0.85). leucoanthocyantin reductase, cinnamic acid 4-hydroxylase, chalcone synthase, dihydroflavonol 4-reductase, flavonol synthase 1, anthocyanidin synthase, anthocyanidin reductase and flavonoid 3’-hydroxylase were the differentially expressed structural genes. Differentially expressed regulator genes: Myb family APL isoform X1, bHLH94-like protein, and bHLH67 were also validated (S6 Table).

FLS1 was highly expressed in Ft, being 7.38-fold higher than in Fes, whereas expressed 4.32-fold in Fea comparing to Fes. Myb family APL isoform X1 and *bHLH*67 showed significant abundance in Fea and Fes versus Ft, whereas bHLH94-like protein was almost 1.9-fold higher in Ft than in Fea and Fes. *CHS* expression was more than 1 -fold higher in Fea versus Ft. *C4H* expression was more than 2-fold higher in both Fes and Fea compared to Ft, while *DFR* expression was about 1.5-fold higher in Fes versus Ft; however, *ANS* expression levels in Fes and Fea were almost the same that is more than 5 -fold in Ft. Taken together, these results suggest that the *FLS1* transcript level may be associated with rutin accumulation in filling stage seeds of buckwheat species.

### Storage proteins involved in the development of *Fagopyrum* spp. seeds

**Fig 7:**
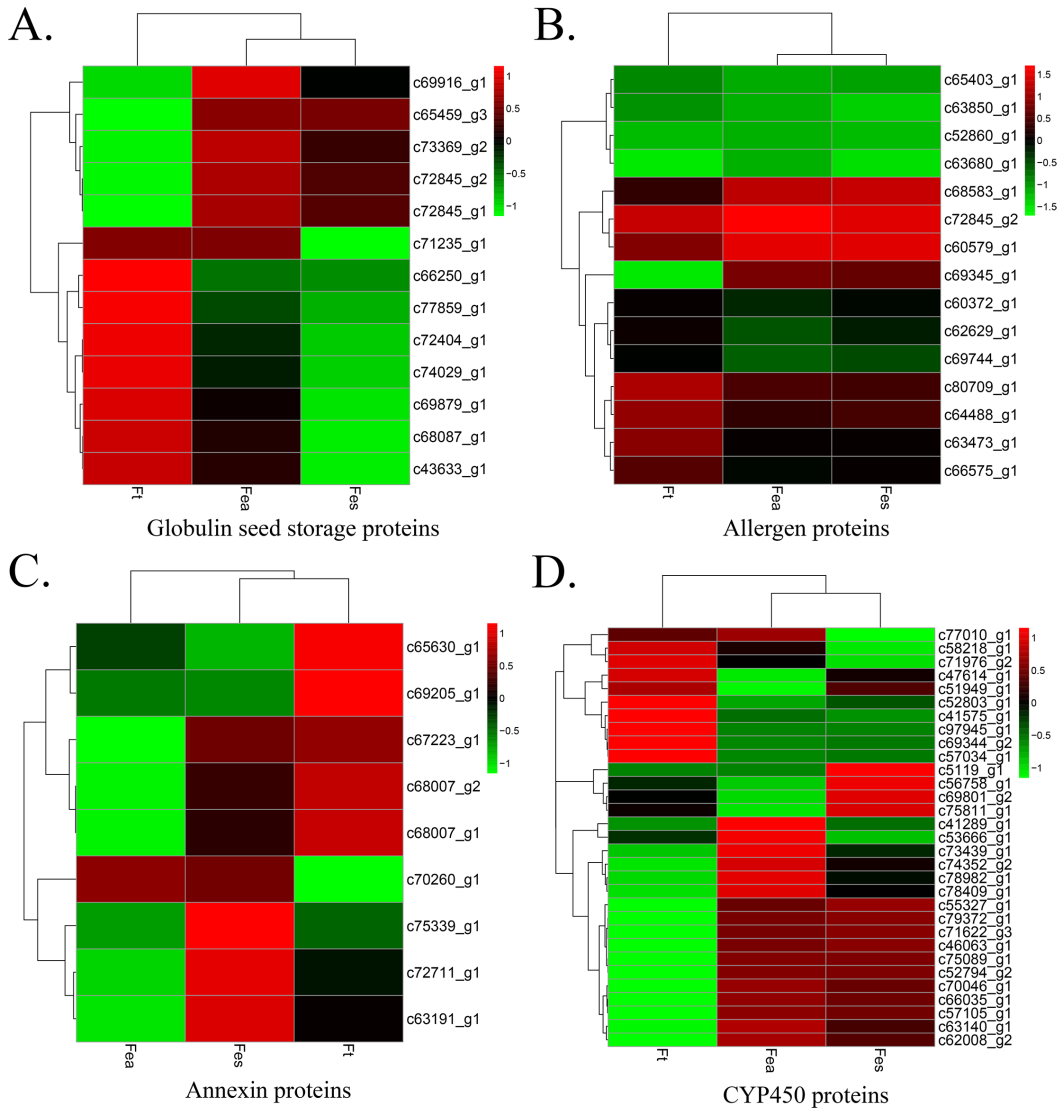
Expression profiles of seed storage proteins and developmental- related CYP450 genes across three species. Clustering analysis of all genes in heatmaps in different columns represent the expression levels in Ft, Fea and Fes. A color scale is shown at the left top. Green color indicates lower expression, while red color indicates higher expression. (A) Expression profile of globulins-related genes. (B) Hierarchical clustering of allergen proteins. (C) Profiling of annexin proteins. (D) Identified *CYP450* genes expression profile.

Seed storage proteins are important for the determination of total protein content in seeds and their quality for various end uses, especially as a dietary protein source. There are four groups of storage proteins: globulins, albumins, prolamins, and glutelins. As a pseudocereal crop, buckwheat seeds have a high content of proteins with balanced ratio and concentrations of essential amino acids. During seed development, storage proteins accumulated. Among them, the globulins are the most abundant ones. 13S legumin-like protein and vicilin-like protein and 2S albumin are three main storage proteins in buckwheat seeds. [20] In the buckwheat transcriptome, 13S globulin seed storage proteins (legumin-like) were annotated with 3 unigenes (c72845_g2, c72845_g1, and c65459_g3), 7S globulin got hits with 9 unigenes (c69879_g1, c68087_g1, c72404_g1, c74029_g1, c71235_g1, c77859_g1, c69916_g1, c73369_g2, and c43633_g1; Fig 7A). legumin A- and B-like proteins each got 1 unigene hit (c73084_g1and c73084_g2, respectively), glutelin type-B 5-like was annotated with 1 unigene (c73921_g1), seed storage 2S albumin superfamily protein was annotated with two unigenes (c47296_g1 and c101651_g1) and vicilin-like protein was annotated with 4 unigenes (c66488_g1, c66488_g2, c74713_g1, and c59598_g1). These genes were highly expressed in Fes and Fea species. There were no hits with the prolamins in any of the buckwheat species. These seed storage proteins are important for seed development and further germination, which could cause some allergic responses in human beings.

Proteins annotated with allergens were searched manually. The pollen allergen MetE from *Amaranthus retroflexus* was expressed in all three species (c80709_g1), and c63473_g1 was annotated as calcium-binding allergen Ole from *B*. *vulgaris* ssp. *vulgaris*. Two unigenes for different 16-kDa allergens were found in Fea, Fes, and Ft (c60579_g1 and c52860_g1), though c52860_g1 had a relatively lower FPKM value. Unigene c68583_g1 annotated as a BW10-kDa allergen protein from Ft; c69345_g1 also annotated as an allergenic protein from Ft, but with a 1000–2000 FPKM level in Fes and Fea and almost 0 FPKM in Ft (Fig 7B).

We also identified proteins that help plants survive against biotic and abiotic stresses. The calcium binding protein, annexin, was found to have 9 annotated unigenes (c72711_g1, c68007_g1, c68007_g2, c6530_g1, c7223_g1, c63191_g1, c69205_g1, c75339_g1, and c70260_g1). Four of them were predicted to be annexin-D8, -D2-like, -D5-like, and annexin -D8-like from *Beta vulgaris* ssp. *vulgaris*, while others were annexin proteins from Ft (c68007_g1, c68007_g2, c72711_g1, and c70260_g1). These annexin proteins have been implicated in a variety of physiological processes in other plants (Fig 7C).

### New genes of P450 family found in seed transcriptome annotation data of buckwheat species

P450 is one of the biggest gene super families [21]. Transcriptome analyses have contributed to the increase in P450 sequences; however, among the annotated genes, most were hypothetical proteins with unknown functions in the present study. Plant cytochrome P450s are implicated in a wide variety of biosynthetic reactions and interact with many biomolecules. Recently, investigations have focused on plant hormones and secondary metabolites, lignin biosynthesis and defensive compounds. The CYP78A5 protein annotated by 2 unigenes (c70046_g1 and c41289_g1) may function at the meristem or organ boundary by regulating directional growth [22]. It may also be required in developing ovules to activate cell proliferation and promote seed growth in association with CYP78A7. CYP76AD1 was annotated by 10 unigenes (c56758_g1, c69801_g2, c47614_g1, c58218_g1, c5119_g1, c55327_g1, c51949_g1, c97945_g1, c89448_g1, and c74352_g2) and may be involved in the betalain biosynthetic pathway, which is part of pigment biosynthesis. CYP82C4 was annotated by 2 unigenes (c52803_g1 and c69344_g2) and may function in the early iron deficiency response. CYP94A1, annotated by 6 unigenes (c75089_g1, c78982_g1, c78409_g1, c53666_g1, c71976_g2, c41575_g1) could catalyze the hydroxylation of various fatty acids and may also be involved in plant defense. CYP714A1, annotated with 2 unigenes (c71622_g3 and c46063_g1), may be involved in the repression of early gibberellin intermediates, and CYP71A1 annotated with 10 unigenes (c73439_g1, c63140_g1, c57034_g1, c66035_g1, c62008_g2, c75811_g1, c52794_g2, c79372_g1, c57105_g1, and c77010_g1) and may function in the development of flavor during seed maturation by acting as *trans*-cinnamic acid 4- hydroxylase (Fig 7D).

### Starch and sucrose metabolism genes

**Fig 8:**
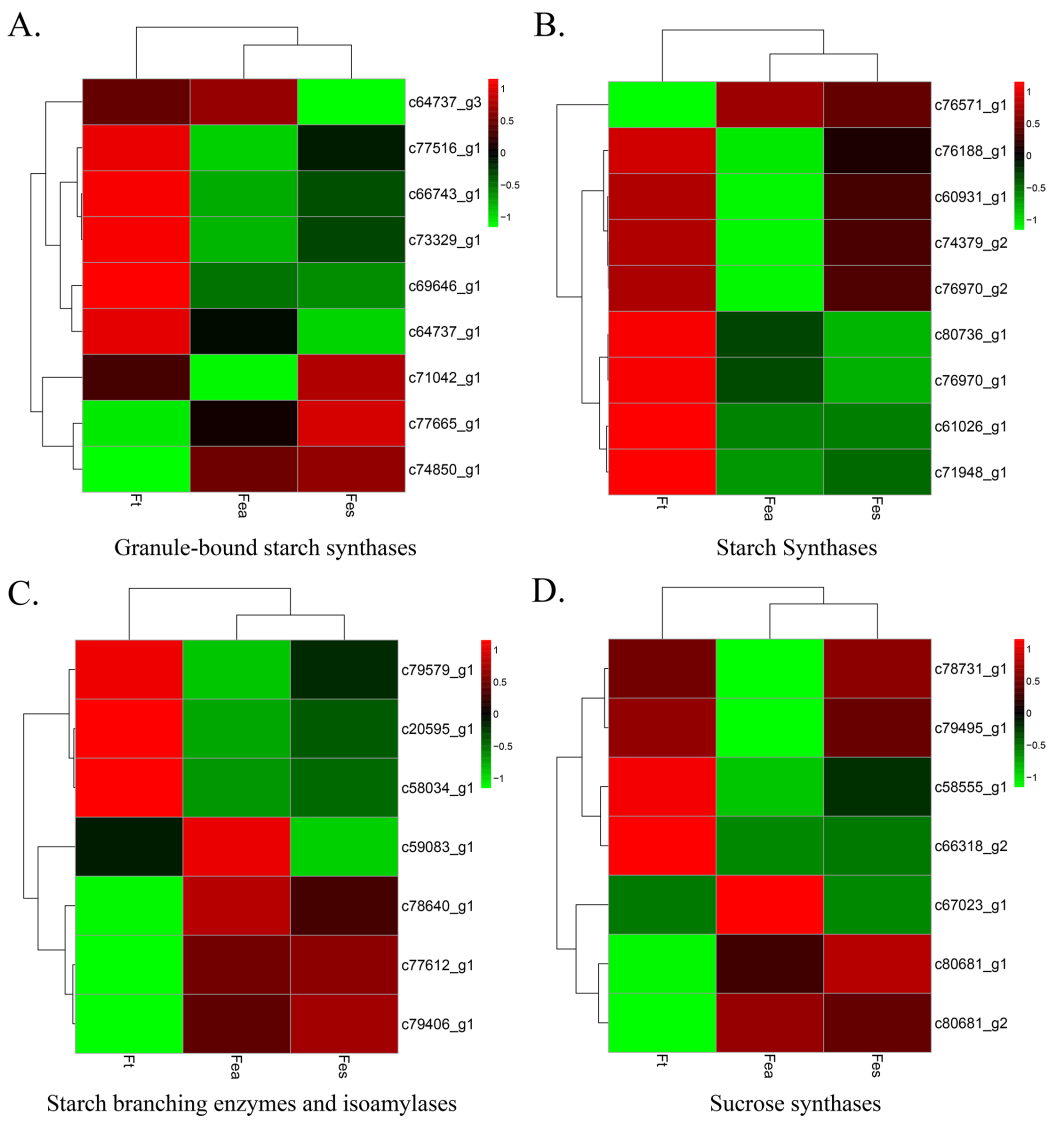
Profiling of carbohydrates metabolism-related genes expressed in filling stage seeds. (A) Granule-bound starch synthases genes (B) Hierarchical clustering of starch synthases. (C) Starch branching enzymes and isoamylases (D) Sucrose synthases. The expression levels all based on Log_2_ normalized FPKM values.

Starch accumulated in the amyloplasts of seeds over a long growing season. Structurally two types of starch are synthesized by different enzymes. In the buckwheat seeds, Granule-bound starch synthase I was annotated with 9 unigenes (c77665_g1, c77516_g1, c74850_g1, c73329_g1, and c64737_g1). c71042_g1 was annotated as granule-bound starch synthase II (Fig 8A). c77612_g1 and c58034_g1 were identified as the ADP-glucose pyrophosphorylase catalytic subunit from *Perilla frutescens*.

Starch synthase (SS) I annotated as c76571_g1, c60931_g1 was annotated as SSII from *A*. *cruentus*, 3 unigenes (c80736_g1, c61026_g1 and c76188_g1) were annotated as SSIII, and 4 unigenes (c74379_g2, c71948_g1, c76970_g1, and c76970_g2) were annotated as SSIV (Fig 8B). In the starch biosynthesis pathway, c59083_g1 and c20595_g1 were annotated as starch branching enzyme I from *Medicago truncatula* and part of starch branching enzyme IIa from *Triticum aestivum*, respectively. c79579_g1 was annotated as an isoamylase1-type starch debrancing enzyme, c79406_g1 was annotated as isoamylase 2 from *B*. *vulgaris* ssp. *vulgaris*, and c78640_g1 was predicted as isoamylase3 from *Citrus sinensis* (Fig 8C).

For the carbohydrate fixation pathway, there is a close correlation between the metabolism of starch and sucrose. Sucrose synthase genes were identified: c78731_g1 had the highest expression and was annotated from *A*. *cruentus* as sucrose synthase1; c58555_g1 and c66318_g2 were also identified as sucrose synthase1, but with 100- and 1000-times lower FPKM values, respectively, compared with c78731_g1. c80681_g2 and c80681_g1 wereas annotated as sucrose synthase 2 isoforms from *A*. *hypochondriacus* and *Eucalyptus grandis*, respectively; c79495_g1 was predicted as sucrose synthase 5 isoform X1 (Fig 8D).

### Transcription factors are important regulators involved in plant development

We used iTAK software to analyze the transcription factors in Fes, Fea and Ft buckwheat species [23]. We identified 2379 unigenes as transcription factors. The main transcription factor families identified were bHLH (107 unigenes), AUX/IAA (50 unigenes), ARF (28 unigenes), WRKY (81 unigenes), MYB (222 unigenes), AP2-EREBP (134 unigenes), NAC (232 unigenes), and MADS (65 unigenes).

## Discussion

*De novo* sequencing methods have been performed on *Fagopyrum* spp. flowers and roots, and the flavonoids biosynthetic pathway-related genes have been discussed [24,15]. In the present study, we sequenced the seed transcriptomes of three *Fagopyrum* spp. by Next Generation RNA-seq, focusing on nutritional properties and rutin content of Ft, Fes and Fea filling-stage seeds was determined. In the filling stage(15-20DAP), RNA transcription activity is more vigorously than in mature seeds [25]. Most flavonoids biosynthesis enzymes are conserved among buckwheat species. In this study we were managing to compare the relative expression levels of unigenes that may influence the final products in flavonoid biosynthesis pathway. We used the pooled data to assemble a uniform reference transcriptome. Then we compared the FPKM value of each candidate unigene. Though the method used in this study has the potential to be improved, the quantitative RT-PCR proved the coherence of the RNA seq results. Enrichment analysis showed that the transcription levels of enzymes in the flavonoid biosynthesis pathway correlated with higher rutin content in developing Fea seeds compared to Fes. However, the DEGs analysis in Ft and Fes did not show obvious enrichment in those flavonoid pathways, which may be attributed to the huge difference in transcript background between these two species. As such, DEGs analysis also showed a lower amount of differentially expressed genes between Fes versus Fea and Fea versus Ft relative to Fes Versus Ft. Hence, Ft and Fes cultivars cannot cross with each other due to their dramatically genome size and structure [26]. Though both are diploid plants, common buckwheat has more repeated sequences and complex chromosomal structures distributing all over its genome [24]. Comparative BLAST analysis of orthologous genes among three buckwheat species showed the evolutionary relationship between Fea and Fes was more closely related than the one between Fes and Ft. These results may pave the way for future breeding strategies to create high rutin content common buckwheat germplasms.

In previous studies, flavonoid level accumulated in the seeds confirmed the correlation between species and the stages of development, as well as metabolic state, and duration and intensity of environmental stresses[27–30]. Furthermore, although there was a fluctuation in rutin content during seed development in all species, the final stage of *Fagopyrum* spp. seed maturation was found to contain different levels of rutin. Our results showed that 20–30 DAP, Ft contained similar amounts of rutin as Fea, whereas Fes rutin content was hardly detected by HPLC. Flavonoid synthase is an important gene family in the biosynthesis pathways. Li *et al*.(2013) reported two kinds of FLS in common buckwheat, FLS1 and FLS2 [31]. Based on the current BLAST results, at least two isoforms of FLS were found in the transcriptome of *Fagopyrum* spp. FLS2 was expressed with almost no difference in all three species (validated by q RT-PCR), and may function as a stress response enzyme[32,33][32][33]. However, FLS1 was highly expressed in both Ft and Fea, whereas Fes had significantly lower expression. As in *Arabidopsis*, only FLS1 functions in flavonoid biosynthesis in a manner analogous to *Fagopyrum* spp. [34]. FLS also controls the divergence of the flow to different products: quercetin and anthocyanin. Anthocyanins and flavonols reportedly share common precursors and that their accumulation pattern is regulated by miR156-targeted SPL, a transcription factor that disrupts the of MYB-bHLH-WD40 complex, leading to accumulation of anthocyanins instead of flavonols [35,36]. These findings showed that dihydroflavonols (dihydrokaempferol and dihydroquercetin) were the same substrates of DFR and FLS1. There should be an equilibrium between these two enzymes as they compete for the same substrate, leading to different products in plants. In crabapple, the ratio between *FLS* and *DFR* transcript abundances determine the color of the leaves; when *McFLS* was overexpressed or *Mc5* was silenced, flavonols production was elevated [37]. The content of pigments in rose, peach, carnatio, zealea, camellia, and petunia flowers were determined, as well as the *FLS* and *DFR* gene expression levels between white and red flowers. FLS and DFR enzymes direct the production of flavonols and anthocyanins, respectively [36]. Elucidating biosynthetic mechanisms will make metabolic engineering of plants easier in order to produce different final products [15, 38]. Current qRT-PCR and FPKM results showed that some enzymes coding for late stage flavonoid biosynthesis were highly expressed in Fes and Fea, especially those involved in proanthocyanin biosynthesis (LAR, DFR, ANS, and ANR). FLS and DFR enzymes may compete for the same substrates to direct synthesis of anthocyanins or flavonols like rutin [36]. Our results showed that *FLS1* expression levels are higher in Fea than Fes, which may play a crucial role in fine tuning of anthocyanin and flavonol biosynthesis.

Despite enzymes involved in metabolism, the majority of buckwheat seed proteins are storage proteins which mainly accumulated in the late embryogenesis stages. The accumulation of storage proteins may influence seed germination. In buckwheat species, the FPKM results indicate that the most abundant unigenes of storage proteins are 13S globulins, vicilin like proteins, BW10KD allergen protein, and legumin A like proteins, legumin B like proteins. Besides, Annexin proteins also expressed highly in Fea and Fes which may function in infection, wounding, and abiotic stress responses. These results indicated that the seed storage protein expression patterns may be different among buckwheat species. Fea shared similar pattern with Fes.

Developing seeds import sucrose, which is cleaved to provide carbon skeletons for the synthesis of other storage compounds [39]. We have detected the sucrose-phosphate synthase and sucrose phosphatase which play key roles in sucrose biosynthesis, whereas the sucrose synthase function in the cleavage of sucrose mainly. In our data, the sucrose synthase transcripts expression levels were much higher in the seeds of three buckwheat species during the filling stage. In contrast, the abundance of enzymes of sucrose biosynthesis are almost 100 times lower than sucrose synthase. The sucrose may be catalyzed by sucrose synthase in the buckwheat seeds to produce UDP-glucose and fructose for the further synthesis of starch in the endosperm. Sucrose also facilitate the storage cell differentiation and reserve accumulation in the developing plant embryos [40].

The granule bound starch synthase and starch synthase gene expression ratio may correlate with the amylose and amylopectin ratio in the seeds such as maize [41]. We have found several GBSS transcripts, c77516_g1 was annotated as *Fagopyrum tataricum* granule bound starch synthase 1, expressed in the level about 100 FPKM in filling stage seeds of three species. While there is a transcript of c77665_g1 annotated as granule bound starch synthase 1 in *Nelumbo nucifera* expressed as 2979 FPKM in FEA, 4354 in Fes and 1808 in Ft which may function in other ways. For the starch synthase 1 (c76571_g1) expressed in the level more than 200 FPKM; starch synthase 2(c60931_g1) expressed at relative much lower levels as around 1 FPKM in those seeds.

Collectively, we sequenced RNA from filling stage seeds from three buckwheat species. The discovery of new genes in buckwheat for the first time in different nutrients pathways will provide further bases and data for future studies. Understanding the molecular regulation of flavonoid biosynthesis in buckwheat species will bolster the breeding and engineering of this plant for different end uses for human beings.

## Supporting information

S1 Fig Length distribution of transcripts and unigenes. (EPS)

S1 Table RNA-sequencing data and statistics of filling-stage buckwheat species seed libraries. (DOCX)

S2 Table *De novo* assembly quality of RNA-sequencing data. (DOCX)

S3 Table Mapping rate of the buckwheat samples to the reference transcriptome assembly. (DOCX)

S4 Table Gene annotation and Blast results against seven public databases of each assembled buckwheat species. (DOCX)

S5 Table Expression profiles of genes involved in flavonoid biosynthesis in buckwheat filling-stage seeds. The bold gene Id were chosen for qRT-PCR experiment.
(DOCX)

S6 Table Validation of differentially expressed genes by Q RT-PCR method: Log_2_Fold change vs.(Log_2_ 2^-ΔΔCt^). (DOCX)
