# Supplementary material for: Transcriptome analysis of filling stage seeds among three buckwheat species with emphasis on rutin accumulation
Source: PLoS One. 2017 Dec 20;12(12):e0189672. doi: 10.1371/journal.pone.0189672 (PMC5738128; doi:10.1371/journal.pone.0189672)
Supplement: S1 Table — (DOCX) [file pone.0189672.s002.docx]

**Table S1.** **RNA-sequencing data and statistics of filling-stage buckwheat species seed libraries.**

| **Table 1. statistics of RNA-seq data of buckwheat species seed libraries** | | | | | | |
| --- | --- | --- | --- | --- | --- | --- |
| sample name | No. of raw reads | No. of clean reads | Clean bases | Q20 (%) | Q30 (%) | GC (%) |
| Fes-R1 | 28163106 | 26681323 | 3.34 | 96.56/93.77 | 93.02/88.58 | 47.27 |
| Fes-R2 | 28947479 | 27568054 | 3.45 | 96.63/93.86 | 93.15/88.73 | 47.04 |
| Fea-R1 | 29681004 | 28929633 | 3.62 | 96.54/93.55 | 93.04/88.26 | 47.93 |
| Fea-R2 | 34126985 | 32293037 | 4.04 | 96.5/93.78 | 92.95/88.63 | 48.01 |
| Ft-R1 | 30457947 | 29891150 | 3.74 | 96.77/94.18 | 93.41/89.29 | 45.95 |
| Ft-R2 | 30260462 | 29591928 | 3.70 | 96.73/94.25 | 93.34/89.38 | 46.01 |

An Illumina 2500 platform was used to sequence the transcriptome of three samples and 125-bp paired-end data were generated for those cDNA libraries. There are two replicates of each species; R1 and R2, replicates 1 and 2, respectively.
