# Supplementary material for: Transcriptome analysis of filling stage seeds among three buckwheat species with emphasis on rutin accumulation
Source: PLoS One. 2017 Dec 20;12(12):e0189672. doi: 10.1371/journal.pone.0189672 (PMC5738128; doi:10.1371/journal.pone.0189672)
Supplement: S2 Table — (DOCX) [file pone.0189672.s003.docx]

**Table S2. *De novo* assembly quality of RNA-sequencing data.**

| **Table 2. *De novo* assembly quality of RNA-seq data** | |
| --- | --- |
| Transcripts generated | 180568 |
| Maximum transcript length | 16709 |
| Minimum transcript length | 201 |
| Average transcript length | 868 |
| Median transcript length | 505 |
| 200-500bp | 89724 |
| 500-1Kb | 38513 |
| 1-2Kb | 33816 |
| ≥ 2Kb | 18515 |
| N50 value | 1468 |
| N90 value | 339 |
