# Supplementary material for: Transcriptome analysis of filling stage seeds among three buckwheat species with emphasis on rutin accumulation
Source: PLoS One. 2017 Dec 20;12(12):e0189672. doi: 10.1371/journal.pone.0189672 (PMC5738128; doi:10.1371/journal.pone.0189672)
Supplement: S3 Table — (DOCX) [file pone.0189672.s004.docx]

**Table S3. Mapping rate of the buckwheat samples to the reference transcriptome assembly.**

| Sample name | Total reads | Total mapped |
| --- | --- | --- |
| Fea_1 | 57859266 | 41497382(71.72%) |
| Fea_2 | 64586074 | 46139290(71.44%) |
| Ft_1 | 59782300 | 44928660(75.15%) |
| Ft_2 | 59183856 | 45851728(77.47%) |
| Fes_1 | 53362646 | 38487490(72.12%) |
| Fes_2 | 55136108 | 39609840(71.84%) |
