# Supplementary material for: Transcriptome analysis of filling stage seeds among three buckwheat species with emphasis on rutin accumulation
Source: PLoS One. 2017 Dec 20;12(12):e0189672. doi: 10.1371/journal.pone.0189672 (PMC5738128; doi:10.1371/journal.pone.0189672)
Supplement: S4 Table — (DOCX) [file pone.0189672.s005.docx]

| **Table S4. Gene annotation and Blast results against seven public databases** | | | | | | |
| --- | --- | --- | --- | --- | --- | --- |
|  | Number of unigenes | | | BLAST hits (%) | | |
|  | Fea | Fes | Ft | Fea | Fes | Ft |
| Annotated in NR | 28331 | 27006 | 25974 | 50.22 | 48.22 | 46.57 |
| Annotated in NT | 10121 | 9902 | 9871 | 17.94 | 17.68 | 17.7 |
| Annotated in KO | 9165 | 8784 | 8592 | 16.24 | 15.68 | 15.4 |
| Annotated in SwissProt | 21558 | 20541 | 19918 | 38.21 | 36.67 | 35.71 |
| Annotated in PFAM | 20618 | 20042 | 19628 | 36.55 | 35.78 | 35.19 |
| Annotated in GO | 22914 | 22198 | 21559 | 40.62 | 39.63 | 38.66 |
| Annotated in KOG | 11381 | 11001 | 10761 | 20.17 | 19.64 | 19.29 |
| Annotated in all databases | 3472 | 3388 | 3362 | 6.15 | 6.04 | 6.02 |
| Annotated in at least one Database | 30496 | 29297 | 28182 | 54.06 | 52.31 | 50.53 |
| Total Unigenes | 56410 | 56001 | 55763 | 100 | 100 | 100 |
