# Supplementary material for: Transcriptome analysis of filling stage seeds among three buckwheat species with emphasis on rutin accumulation
Source: PLoS One. 2017 Dec 20;12(12):e0189672. doi: 10.1371/journal.pone.0189672 (PMC5738128; doi:10.1371/journal.pone.0189672)
Supplement: S5 Table — The bold gene Id were chosen for qRT-PCR experiment. (DOCX) [file pone.0189672.s006.docx]

| **Gene ID** | **Length(bp)** | **E-value** | **FPKM_Fea_1** | **FPKM_Fea_2** | **FPKM_Fes_1** | **FPKM_Fes_2** | **FPKM_Ft_1** | **FPKM_Ft_2** |
| --- | --- | --- | --- | --- | --- | --- | --- | --- |
| **PHENYLALANINE AMMONIA-LYASE** | | | | | | | | |
| **c78103_g1** | 3712 | 0 | 222.38 | 241.28 | 147.09 | 140.57 | 114.33 | 142.69 |
| c76752_g1 | 1930 | 0 | 55.24 | 109.88 | 65.4 | 63.72 | 48.01 | 69.79 |
| c65505_g1 | 1649 | 0 | 54.93 | 43.26 | 31.69 | 29.08 | 51.63 | 109.83 |
| c65563_g1 | 1180 | 7.45E-155 | 70.81 | 45.49 | 29.92 | 26.92 | 13.27 | 21.55 |
| c43581_g1 | 452 | 4.22E-51 | 57.09 | 45.04 | 16.9 | 18.3 | 51.2 | 124.61 |
| c80029_g1 | 3292 | 0 | 41.17 | 29.25 | 19.89 | 19.07 | 18.07 | 38.68 |
| **CINNAMATE 4-HYDROXYLASE** | | | | | | | | |
| c78622_g1 | 2012 | 0 | 351.82 | 326.31 | 165.07 | 164.18 | 118.65 | 157.63 |
| **c65841_g1** | 1759 | 0 | 38.32 | 16.76 | 23.58 | 25.25 | 2.74 | 7.1 |
| **4-COUMARATE:COA LIGASE** | | | | | | | | |
| c79904_g1 | 2302 | 0 | 110.16 | 83.16 | 62.94 | 63.53 | 39.61 | 71.7 |
| c75279_g1 | 2248 | 0 | 23.69 | 22.1 | 19.6 | 19.8 | 32.23 | 49.82 |
| c80099_g1 | 1968 | 0 | 37.8 | 21.51 | 23.08 | 23.07 | 20.22 | 18.17 |
| c79681_g1 | 1954 | 0 | 19.32 | 19.18 | 12.91 | 13.04 | 7.14 | 6.21 |
| **c76157_g1** | 3528 | 0 | 1621.28 | 1313.85 | 1563.72 | 1526.21 | 1529.67 | 1279.27 |
| **FLAVONOID 3',5'-HYDROXYLASE** | | | | | | | | |
| **c79279_g1** | 1787 | 0 | 18.56 | 18.67 | 9.88 | 10 | 10.2 | 7.63 |
| **FLAVONOID 3'-HYDROXYLASE** | | | | | | | | |
| **c75726_g2** | 1730 | 0 | 121.42 | 250.25 | 232.39 | 225.84 | 6.09 | 6.06 |
| c67918_g1 | 1716 | 1.60E-179 | 8.16 | 6.3 | 4.6 | 4.89 | 24.37 | 9.41 |
| c79262_g1 | 1971 | 0 | 74.57 | 63.73 | 55.18 | 51.9 | 26.43 | 20.22 |

**S5 Table.** Expression profiles of genes involved in flavonoid biosynthesis in buckwheat filling-stage seeds.

The bold gene Id were chosed for qRT-PCR experiment.

**S5 Table. Continued**

| **Gene ID** | **Length(bp)** | **E-value** | **FPKM_Fea_1** | **FPKM_Fea_2** | **FPKM_Fes_1** | **FPKM_Fes_2** | **FPKM_Ft_1** | **FPKM_Ft_2** |
| --- | --- | --- | --- | --- | --- | --- | --- | --- |
| **FLAVANONE-3-HYDROXYLASE** | | | | | | | | |
| **c74521_g1** | 1273 | 0 | 23.86 | 34.14 | 30.72 | 30.62 | 7.36 | 10.36 |
| c76122_g1 | 1851 | 7.86E-126 | 20.28 | 20.23 | 16.32 | 14.5 | 3.66 | 1.54 |
| **CHALCONE SYNTHASE** | | | | | | | | |
| **c70318_g5** | 1019 | 5.78E-161 | 585.44 | 423.81 | 330.89 | 310.92 | 79.95 | 70.46 |
| c70318_g2 | 887 | 1.30E-108 | 320.52 | 320.85 | 221.67 | 205.09 | 210.66 | 199.39 |
| c70318_g4 | 949 | 1.72E-172 | 123.07 | 140.48 | 37.25 | 41.22 | 65.71 | 47.77 |
| c70318_g1 | 547 | 1.11E-110 | 137.78 | 190.28 | 69.89 | 63.5 | 44.31 | 34.32 |
| c70318_g6 | 873 | 1.79E-137 | 193.75 | 125.98 | 118.69 | 112.94 | 110.77 | 122.64 |
| c70318_g3 | 610 | 3.46E-119 | 204.84 | 194.37 | 160.63 | 153.2 | 98.96 | 112.67 |
| **CHALCONE ISOMERASE** | | | | | | | | |
| c67312_g1 | 1111 | 2.82E-174 | 113.68 | 83.55 | 66.58 | 73.62 | 62.37 | 79.05 |
| **DIHYDROFLAVONOL-4-REDUCTASE** | | | | | | | | |
| **c62371_g1** | 1543 | 0 | 32.17 | 35.35 | 10.58 | 10.46 | 96.13 | 83.35 |
| c75529_g1 | 1259 | 1.86E-165 | 10.77 | 11.37 | 11.87 | 12.51 | 18.2 | 11.39 |
| c70955_g1 | 1349 | 2.53E-143 | 18.55 | 19.66 | 19.84 | 21.21 | 3.53 | 4.11 |
| **FLAVONOL SYNTHASE** | | | | | | | | |
| **c71512_g1** | 1292 | 0 | 49.86 | 52.74 | 49.12 | 47.74 | 48.54 | 59.13 |
| **c62983_g1** | 1409 | 0 | 53.58 | 39.02 | 3.89 | 2.77 | 149.94 | 152.56 |
| c62154_g1 | 515 | 4.43E-27 | 5.63 | 4.16 | 7.6 | 6.66 | 1.42 | 2.8 |
| **ANTHOCYANIDIN SYNTHASE** | | | | | | | | |
| **c69216_g1** | 1702 | 0 | 148.94 | 213.61 | 127.52 | 122.62 | 25.83 | 38.32 |

**S5 Table. Continued**

| **Gene ID** | **Length(bp)** | **E-value** | **FPKM_Fea_1** | **FPKM_Fea_2** | **FPKM_Fes_1** | **FPKM_Fes_2** | **FPKM_Ft_1** | **FPKM_Ft_2** |
| --- | --- | --- | --- | --- | --- | --- | --- | --- |
| **ANTHOCYANIDIN REDUCTASE** | | | | | | | | |
| **c71792_g1** | 1534 | 0 | 311.52 | 310.1 | 172.88 | 162.64 | 89.91 | 144.7 |
| **LEUCOANTHOCYANTIN REDUCTASE** | | | | | | | | |
| **c73957_g1** | 3141 | 0 | 170.05 | 116.21 | 57.9 | 57.89 | 19.48 | 23.88 |
