# Supplementary material for: Transcriptome analysis of filling stage seeds among three buckwheat species with emphasis on rutin accumulation
Source: PLoS One. 2017 Dec 20;12(12):e0189672. doi: 10.1371/journal.pone.0189672 (PMC5738128; doi:10.1371/journal.pone.0189672)
Supplement: S6 Table — (DOCX) [file pone.0189672.s007.docx]

**S6 Table. Validation of differentially expressed genes by Q RT-PCR method: Log_2_Fold Change vs.（Log_2_ 2^^-δδCt^）**

| **Gene ID** | **Fes vs. Ft** | **Fea vs. Ft** | **Fea vs. Fes** | **Annotation** | **Primers** |
| --- | --- | --- | --- | --- | --- |
| c74254_g1 | 1.66（1.44） | 2.53（2.43） | NA | bHLH67 | 5’GGAGAGGAGGAAAAGGAAGAGAAC3’  5’CAGAAGCTGCTCTAGCTCCTTCAC3’ |
| c66126_g1 | -2.12（-1.87） | -2.73（-1.85） | NA | bHLH94-like | 5’CAAAGAGGAAATCGAAAACCAGAG3’  5’ATGGTTGAGTACTGTGGGAAGCTG3’ |
| c63710_g1 | 2.60（3.77） | 2.59（4.02） | NA | myb family APL isoform X1 | 5’CGTACTCACAACCGACCCCAAGCC3’  5’CGTTGAATTCCTTGTGTGGCTGCT3’ |
| c73957_g1 | NA | NA | 1.43（1.56） | LAR | 5’TTCATTGGCCGGTTTGTGACGGAG3’  5’GCCTCCGACGACGGATATGACAAC3’ |
| c65841_g1 | 2.78（2.92） | 3.17（2.24） | NA | C4H | 5’CGATCTCAACCACCGCAATCTCAC3’  5’ACCGTAAACAGTAAACACCATATC3’ |
| c78103_g1 | NA | 1.35(1.09) | NA | CHS | 5’TACCCCGACTACTACTTCAGG3’  5’ATGACGTGGGTGATCTTGGACTTG3’ |
| c62983_g1 | -5.27(-7.38) | NA | 4.03(4.32) | FLS1 | 5’GACACAATCCCGCTTGAGTAT3’  5’AAACTCCTTTCCGACCCTC3’ |
| c74632_g1 | 1.23(1.49) | 1.80(1.93) | NA | DFR | 5’TGCCAAGTTTCCCTCCAAGTC3’  5’CTCGGGATACTTGTTTCGGAGC3’ |
| c69216_g1 | 2.20 (5.77) | 2.94(5.46) | NA | ANS | 5’GAGTACGTGAGGCCGGAAGAGGAG3’  5’ACCCCATTCCATGGCAGCCTTCTT3’ |
| c79262_g1 | 1.43 (1.72) | 1.99 (2.20) | NA | F3'H | 5’AACATCCCCAAGAACGCCAC3’  5’GCGGTAAGAAACTGGACCATC3’ |
| c71792_g1 | 0.91 (0.43) | 1.20(1.64) | 1.12 (1.89) | ANR | 5’GTCAACACCACCGTTAGAGATCCT3’  5’GCTGGCTTGATCATATCATTCTCT3’ |
